# Natriuretic peptides modulate ATP-sensitive K^+^ channels in rat ventricular cardiomyocytes

**DOI:** 10.1007/s00395-014-0402-4

**Published:** 2014-01-30

**Authors:** Dwaine S. Burley, Charles D. Cox, Jin Zhang, Kenneth T. Wann, Gary F. Baxter

**Affiliations:** Cardiff School of Pharmacy and Pharmaceutical Sciences, Cardiff University, King Edward VII Avenue, Cardiff, CF10 3NB UK

**Keywords:** Natriuretic peptides, Cardiomyocytes, Electrophysiology, Ion channels

## Abstract

B-type natriuretic peptide (BNP) and C-type natriuretic peptide (CNP), and (Cys-18)-atrial natriuretic factor (4–23) amide (C-ANF), are cytoprotective under conditions of ischemia–reperfusion, limiting infarct size. ATP-sensitive K^+^ channel (K_ATP_) opening is also cardioprotective, and although the K_ATP_ activation is implicated in the regulation of cardiac natriuretic peptide release, no studies have directly examined the effects of natriuretic peptides on cardiac K_ATP_ activity. Normoxic cardiomyocytes were patch clamped in the cell-attached configuration to examine sarcolemmal K_ATP_ (sK_ATP_) activity. The K_ATP_ opener pinacidil (200 μM) increased the open probability of the patch (NPo; values normalized to control) at least twofold above basal value, and this effect was abolished by HMR1098 10 μM, a selective K_ATP_ blocker (5.23 ± 1.20 versus 0.89 ± 0.18; *P* < 0.001). We then examined the effects of BNP, CNP, C-ANF and 8Br-cGMP on the sK_ATP_ current. Bath application of BNP (≥10 nM) or CNP (≥0.01 nM) suppressed basal NPo (BNP: 1.00 versus 0.56 ± 0.09 at 10 nM, *P* < 0.001; CNP: 1.0 versus 0.45 ± 0.16, at 0.01 nM, *P* < 0.05) and also abolished the pinacidil-activated current at concentrations ≥10 nM. C-ANF (≥10 nM) enhanced K_ATP_ activity (1.00 versus 3.85 ± 1.13, at 100 nM, *P* < 0.05). The cGMP analog 8Br-cGMP 10 nM dampened the pinacidil-activated current (2.92 ± 0.60 versus 1.53 ± 0.32; *P* < 0.05). Natriuretic peptides modulate sK_ATP_ current in ventricular cardiomyocytes. This may be at least partially associated with their ability to augment intracellular cGMP concentrations via NPR-A/B, or their ability to bind NPR-C with high affinity. Although the mechanism of modulation requires elucidation, these preliminary data give new insights into the relationship between natriuretic peptide signaling and sK_ATP_ in the myocardium.

**Electronic supplementary material:**

The online version of this article (doi:10.1007/s00395-014-0402-4) contains supplementary material, which is available to authorized users.

## Introduction

The natriuretic peptides are a family of structurally related mediators with diverse autocrine/paracrine and endocrine functions in multiple tissues but they are especially involved in cardiovascular homeostasis [6, 31]. In the circulation, C-type natriuretic peptide (CNP), which is predominantly of vascular origin under normal physiological conditions, exerts autocrine/paracrine actions that are well characterized in the vessel wall [33]. The cardiac-derived atrial natriuretic peptide (ANP) and B-type natriuretic peptide (BNP) exert pressure- and volume-regulating roles, which may be viewed as classic endocrine functions [31]. However, there is extensive evidence that ANP and BNP also exert multiple autocrine/paracrine actions within cardiac tissue [6]. These local cardiac actions may be particularly important under pathological conditions when there is enhanced release of ANP and BNP from tissue stores [40]. These include conditions associated with pressure or volume overload, cardiac remodeling and hypoxia where the peptides may exert fundamental (counter-) regulatory actions within myocardium [17, 28, 40, 45].

Limited pharmacological evidence suggests a role of ATP-sensitive K^+^ channel (K_ATP_) opening since protection is lost in the presence of the channel blockers glibenclamide and sodium 5-hydroxydecanoate [5]. This latter mechanism is little understood. Of particular interest, in pancreatic islet β cells, ANP exerts an insulinotrophic action, associated with K_ATP_ blockade [36, 43]. Furthermore, inwardly rectifying K^+^ channel 6.2 (K_ir_6.2) deficient mice were demonstrated to be more susceptible to stretch-induced ANP release compared to wild type, suggesting a negative feedback axis between K_ATP_ and cardiac natriuretic peptide release [38]. These findings are intriguing as they suggest a plausible regulatory relationship between natriuretic peptides and K_ATP_ in distinct endocrine secretory glands and specialized endocrine organs [36, 38]. It is noteworthy that both cardiac and pancreatic K_ATP_ contain the K_ir_6.2 core. However, they differ with respect to the sulphonylurea receptor (SUR), with K_ir_6.2 coupled to SUR2A in cardiomyocytes and to SUR1 in pancreatic beta cells 1:1 tetrameric stoichiometry [1, 39].

In view of the increasing interest in the roles and therapeutic potential of natriuretic peptides in cardiac disease, it is important to characterize the actions of natriuretic peptides on K_ATP_ function in cardiomyocytes. As such, this study provides the first comprehensive and comparative electrophysiological investigation of natriuretic peptides on cardiac sarcolemmal K_ATP_ (sK_ATP_) activity. After characterizing sK_ATP_ activity in adult rat ventricular cardiomyocytes, we sought to test the hypothesis that natriuretic peptides promote sK_ATP_ opening by observing the effects of BNP and CNP together with the natriuretic peptide clearance receptor (NPR-C) agonist (Cys-18)-atrial natriuretic factor (4–23) amide (C-ANF) on sK_ATP_ activity in these cells. Our data provide a characterization of the actions of natriuretic peptides on sK_ATP_. They strongly suggest that, rather than activating sK_ATP_, BNP and CNP at physiological concentrations, and at supraphysiological concentrations relevant to circulating plasma levels in cardiac disease and therapeutic use, inhibit the ion channel. They also suggest that the inhibition seen with BNP and CNP is not due to NPR-C agonism because C-ANF did not depress sK_ATP_ activity.

## Methods

### Cardiomyocyte isolation

We used a total of 64 adult male Sprague–Dawley rats (270–350 g, Harlan Laboratories Bicester, Oxford) for this study. Their care and use were in accordance with UK Home Office Guidelines on the Animals (Scientific Procedures) Act 1986 (The Stationary Office, London, UK). Following pentobarbital anesthesia, hearts were excised and left ventricular cardiac myocytes were isolated using a standard enzymatic digestion protocol. Myocytes were seeded at a density of 20,000 rods/well on extracellular matrix gel-coated plastic coverslips, and cultured overnight under normal CO_2_ incubator conditions at 37 °C, prior to treatments and patch clamping. See online resource for full details.

### Electrophysiology

The bath solution was in mM: 150 NaCl, 3 KCl, 10 d-glucose, 10 HEPES, pH 7.2. The recording pipette contained in mM: 5 NaCl, 140 KCl, 1 MgCl_2_, 1 CaCl_2_, 11 EGTA, 10 HEPES, pH 7.2. During the sK_ATP_ channel characterization phase of this study (see series 1), pipette solutions containing KCl 70 mM:NaCl 70 mM (NaCl, 70 mM equimolar substitution) and KCl 200 mM were used as comparator to the standard pipette solution. An Axon CV-4 patch clamp headstage (Axon Instruments, USA) was mounted on a three axis hydraulic micromanipulator (Narashige, Japan). Signals were amplified using an Axopatch 1D patch clamp amplifier (Axon Instruments, USA) and Neurolog DC amplifier (Digitimer Ltd., UK), and digitized using a National Instruments BNC 2110 digitizer. Signals were typically filtered at 5 kHz and sampling rate was 20 kHz. Signals were visualized on an OX722 METRIX oscilloscope (ITT instruments) or computer screen.

Electrodes were pulled from filamented borosilicate glass capillaries (1.5/0.86 OD and ID, respectively; Harvard Apparatus, UK) and fine polished using a DMZ Universal Puller (Zeitz-Instrumente, Germany). Microelectrodes had resistances of 5–10 MΩ.

Single channel recordings were made from cell-attached patches, and performed at room temperature 22–24 °C, as our setup does not contain a Peltier thermoelectric device for cooling and heating. The electrophysiological gating properties of adult rat cardiac K_ATP_ do not significantly change at temperatures ranging 20–30 °C [26]. In an independent study, Kohlhardt and colleagues observed a consistent but slight increase in neonatal rat cardiac K_ATP_ activity at temperatures ranging 29–39 °C compared to 19–29 °C [21].

Following gigaohm seal formation, currents passing through single ion channels were observed and recorded. Recordings (45 s) were made over a range of patch potentials: 0, −30, −60, −90, and −120 mV. The parameter NPo, where *N* is the number of channels in the patch and Po the open probability of one channel, was used to determine the effects of different compounds and natriuretic peptides on K_ATP_ activity. Po is derived from the sum of individual channel opening times (*O*) and individual closed times (*C*), thus Po = *O*/(*O* + *C*). WinEDR v3.2.2 software (Strathclyde University, UK) was used for data acquisition and single channel analysis.

### Materials

Rat BNP-(1–32) and C-ANF-(4–23) were obtained from Sigma-Aldrich UK, and both CNP-(1–22) and 8Br-cGMP from Tocris bioscience UK. sK_ATP_ channel opener pinacidil (Sigma-Aldrich, UK) was dissolved in dimethylsulfoxide (DMSO; maximal final concentration of 0.25 % v/v). HMR1098, a selective sK_ATP_ inhibitor, was the kind gift of Dr Jürgen Pünter, Sanofi-Aventis Germany. With the exception of HMR1098, all compounds were diluted in bath solution (see recording solutions); HMR1098 was diluted in unsupplemented medium 199.

### Treatments

The number of cells patched is shown in brackets. All cells from group 4 onwards were patched with KCl 140 mM in the patch pipette. All treatments were randomized.

### Series 1

In these experiments, we sought to characterize the ion selectivity and conductance of sK_ATP_, thus cells were patched using different concentrations of KCl with or in the absence of NaCl, which was used as an equimolar substitute (group 1–3). In addition, long established and experimentally characterized K_ATP_ modulators were used to pharmacologically test whether the channel observed in our patch clamp recordings is sK_ATP_ (group 4–7).

#### Ion selectivity experiments

Group 1, KCl 70 mM and NaCl 70 mM (*n* = 4)

Group 2, KCl 140 mM (*n* = 5)

Group 3, KCl 200 mM (*n* = 4)

#### sK_ATP_ channel modulation experiments

Group 4, control (*n* = 12): cells were pretreated with unsupplemented medium 199 for 30 min, then patch clamped in bath solution, or in bath solution containing DMSO 0.25 % v/v.

Group 5, pinacidil 200 μM (*n* = 7): cells were pretreated with unsupplemented medium 199 for 30 min, then patch clamped following bath application of pinacidil.

Group 6, HMR1098 10 μM (*n* = 8): cells were pretreated with HMR1098 in unsupplemented medium 199 for 30 min, then patch clamped in bath solution, or in bath solution containing DMSO 0.25 % v/v.

Group 7, HMR1098 + pinacidil (*n* = 6): cells were pretreated with HMR1098 in unsupplemented medium 199 for 30 min, then patch clamped following bath application of pinacidil.

### Series 2

These experiments were designed to examine the effect of natriuretic peptides on sK_ATP_ channel activity and conductance. Cells were patched clamped in bath solution containing BNP, CNP or C-ANF, in the absence or in the presence of pinacidil. All natriuretic peptides were applied at six concentrations ranging from 0.01 to 1,000 nM. Two independent sets of experiments were done for low concentrations (0.01, 0.1 and 1 nM) and high concentrations (10, 100 and 1,000 nM) of BNP and CNP, with each series having their own separate control and pinacidil treatment groups, respectively. Experiments with C-ANF were done as a single set.

#### The effect of BNP on sK_ATP_ activity

Set 1: the effect of low concentrations of BNP on sK_ATP_ activity

Group 8, control (*n* = 8): cells were patched clamped in bath solution, or in bath solution containing DMSO 0.25 % v/v.

The following compounds were bath applied:

Group 9, pinacidil 200 μM (*n* = 10)

Group 10, BNP 0.01 nM (*n* = 5)

Group 11, BNP 0.1 nM (*n* = 3)

Group 12, BNP 1 nM (*n* = 5)

Group 13, BNP 0.01 nM + pinacidil (*n* = 3)

Group 14, BNP 0.1 nM + pinacidil (*n* = 5)

Group 15, BNP 1 nM + pinacidil (*n* = 3)

Set 2: the effect of high concentrations of BNP on sK_ATP_ activity

Group 16, control (*n* = 35): cells were patched clamped in bath solution, or in bath solution containing DMSO 0.25 % v/v.

The following compounds were bath applied:

Group 17, pinacidil 200 μM (*n* = 41)

Group 18, BNP 10 nM (*n* = 9)

Group 19, BNP 100 nM (*n* = 8)

Group 20, BNP 1,000 nM (*n* = 14)

Group 21, BNP 10 nM + pinacidil (*n* = 9)

Group 22, BNP 100 nM + pinacidil (*n* = 8)

Group 23, BNP 1,000 nM + pinacidil (*n* = 10)

#### The effect of CNP on sK_ATP_ activity

Set 1: the effect of low concentrations of CNP on sK_ATP_ activity

Group 24, control (*n* = 5): cells were patched clamped in bath solution, or in bath solution containing DMSO 0.25 % v/v.

The following compounds were bath applied:

Group 25, pinacidil 200 μM (*n* = 4)

Group 26, CNP 0.01 nM (*n* = 3)

Group 27, CNP 0.1 nM (*n* = 3)

Group 28, CNP 1 nM (*n* = 3)

Group 29, CNP 0.01 nM + pinacidil (*n* = 4)

Group 30, CNP 0.1 nM + pinacidil (*n* = 3)

Group 31, CNP 1 nM + pinacidil (*n* = 4)

Set 2: the effect of high concentrations of CNP on sK_ATP_ activity

Group 32, control (*n* = 29): cells were patched clamped in bath solution, or in bath solution containing DMSO 0.25 % v/v.

The following compounds were bath applied:

Group 33, pinacidil 200 M (*n* = 34)

Group 34, CNP 10 nM (*n* = 10)

Group 35, CNP 100 nM (*n* = 9)

Group 36, CNP 1,000 nM (*n* = 16)

Group 37, CNP 10 nM + pinacidil (*n* = 8)

Group 38, CNP 100 nM + pinacidil (*n* = 8)

Group 39, CNP 1,000 nM + pinacidil (*n* = 8)

#### The effect of low and high concentrations of C-ANF on sK_ATP_ activity

Group 40, control (*n* = 7): cells were patched clamped in bath solution, or in bath solution containing DMSO 0.25 % v/v.

The following compounds were bath applied:

Group 41, pinacidil 200 μM (*n* = 11)

Group 42, C-ANF 0.01 nM (*n* = 6)

Group 43, C-ANF 0.1 nM (*n* = 3)

Group 44, C-ANF 1 nM (*n* = 5)

Group 45, C-ANF 0.01 nM + pinacidil (*n* = 7)

Group 46, C-ANF 0.1 nM + pinacidil (*n* = 4)

Group 47, C-ANF 1 nM + pinacidil (*n* = 7)

Group 48, C-ANF 10 nM (*n* = 5)

Group 49, C-ANF 100 nM (*n* = 4)

Group 50, C-ANF 1,000 nM (*n* = 5)

Group 51, C-ANF 10 nM + pinacidil (*n* = 4)

Group 52, C-ANF 100 nM + pinacidil (*n* = 4)

Group 53, C-ANF 1,000 nM + pinacidil (*n* = 6)

### Series 3

cGMP generation is the common second messenger signal following receptor stimulation by BNP and CNP. These experiments examined if 8Br-cGMP, a cell-permeable analog of cGMP, could mimic the effects of these peptides.

#### The effect of cGMP on sK_ATP_ activity

Group 54, control (*n* = 18): cells were patched clamped in bath solution, or in bath solution containing DMSO 0.25 % v/v.

The following compounds were bath applied:

Group 55, pinacidil 200 μM (*n* = 12)

Group 56, 8Br-cGMP 10 nM (*n* = 10)

Group 57, 8Br-cGMP + pinacidil (*n* = 14)

### PCR and western blotting

Gene and protein expression of K_ATP_ channel subunits were determined in myocardial tissue extracts by RT-PCR and Western blotting. See online resource for full descriptions.

### Data analysis

Data are expressed as mean ± standard error of the mean (SEM). Ion channel open probabilities (NPo) are normalized to control. Raw data corresponding to specific treatment groups were compared for statistical significance using Dunnett’s or Newman-Keuls’ multiple comparison tests following one-way analysis of variance (ANOVA). Differences between arithmetic means were considered significant when *P* < 0.05. Data were analyzed using GraphPad Prism 5 software (GraphPad software Inc., USA).

## Results

### sK_ATP_ channel composition revealed by PCR and Western blotting

**Fig. 1:**
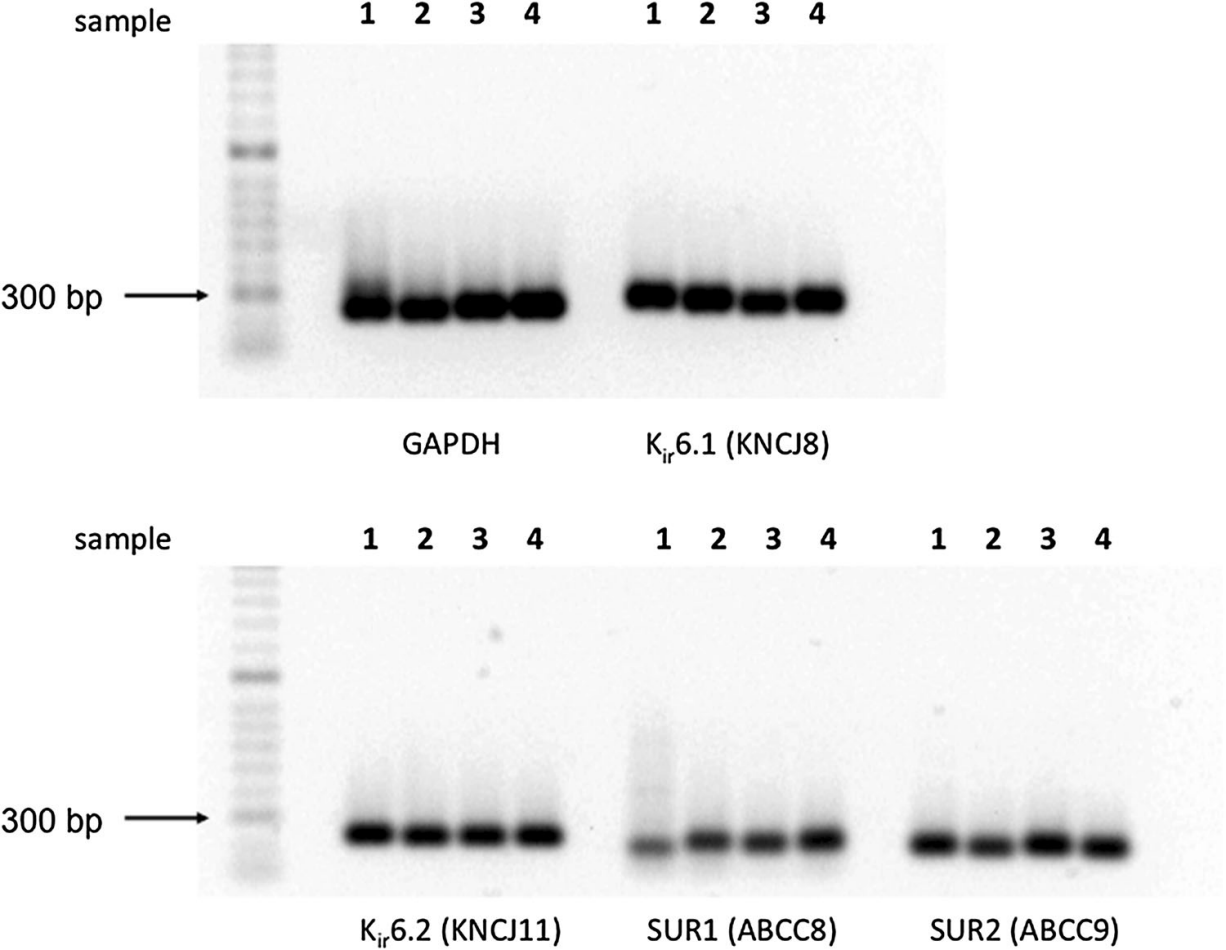
RT PCR amplification of GAPDH and K_ATP_ pore forming and receptor subunit mRNA. Samples are from four independent cardiomyocyte isolations from rat left ventricle. The gene coding for each K_ATP_ subunit is clearly seen. All samples were diluted in Novel Juice (Genedirex, USA), a non-mutagenic nucleic acid stain, and were separated on the same 15 by 25 cm 1 % agarose gel for 6 h prior to UV transillumination and photo aquisition. The following PCR products were obtained, and the gene and product size is shown in brackets: GAPDH (223 bp), K_ir_6.1 (KNCJ8, 227 bp), K_ir_6.2 (KNCJ11, 201 bp), SUR1 (ABCC8, 169 bp) and SUR2 (ABCC9, 228 bp)

**Fig. 2:**
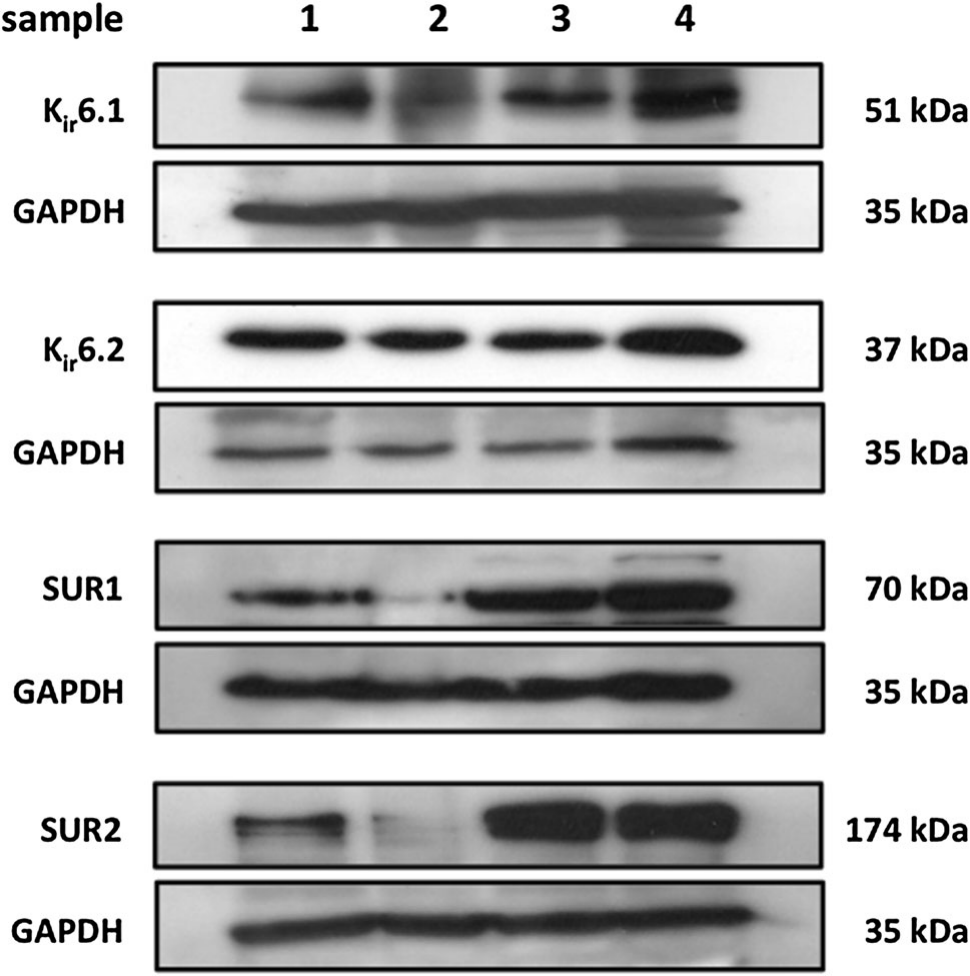
Western blots showing the protein expression of the K_ATP_ pore forming and receptor subunits in cardiomyocytes isolated from left ventricle. Samples consist of protein extracted from the same four independent cardiomyocyte isolations as mentioned in the legend for Fig. 1. Strong K_ir_6.2, SUR1 and SUR2 protein expression is seen, whereas K_ir_6.1 expression is weak comparably. A 70 kDa band is seen for SUR1 and not the predicted 177 kDa, but according to Pu and colleagues [32], this could be a SUR1 short form splice variant. The following amount of protein was loaded when probing for each K_ATP_ subunit: 150 μg for K_ir_6.1, 80 μg for K_ir_6.2, and 100 μg for SUR1 and SUR2

We confirmed the expression of all K_ATP_ subunits at the gene and protein level (Figs. 1, 2) in at least three out of four ventricular myocardial samples analyzed. Strong gene expression was evident for all subunits in all samples analyzed; furthermore, K_ir_6.1, K_ir_6.2, SUR1 and SUR2 subunit proteins were strongly expressed. These expression patterns confirm the presence of all K_ATP_ subunit proteins in the cardiomyocyte and indicate the possibility that alternative K_ATP_ subunit configurations might be present in the cardiac sarcolemma alongside the native K_ir_6.2/SUR2A channel.

### sK_ATP_ electrophysiological characterization

**Fig. 3:**
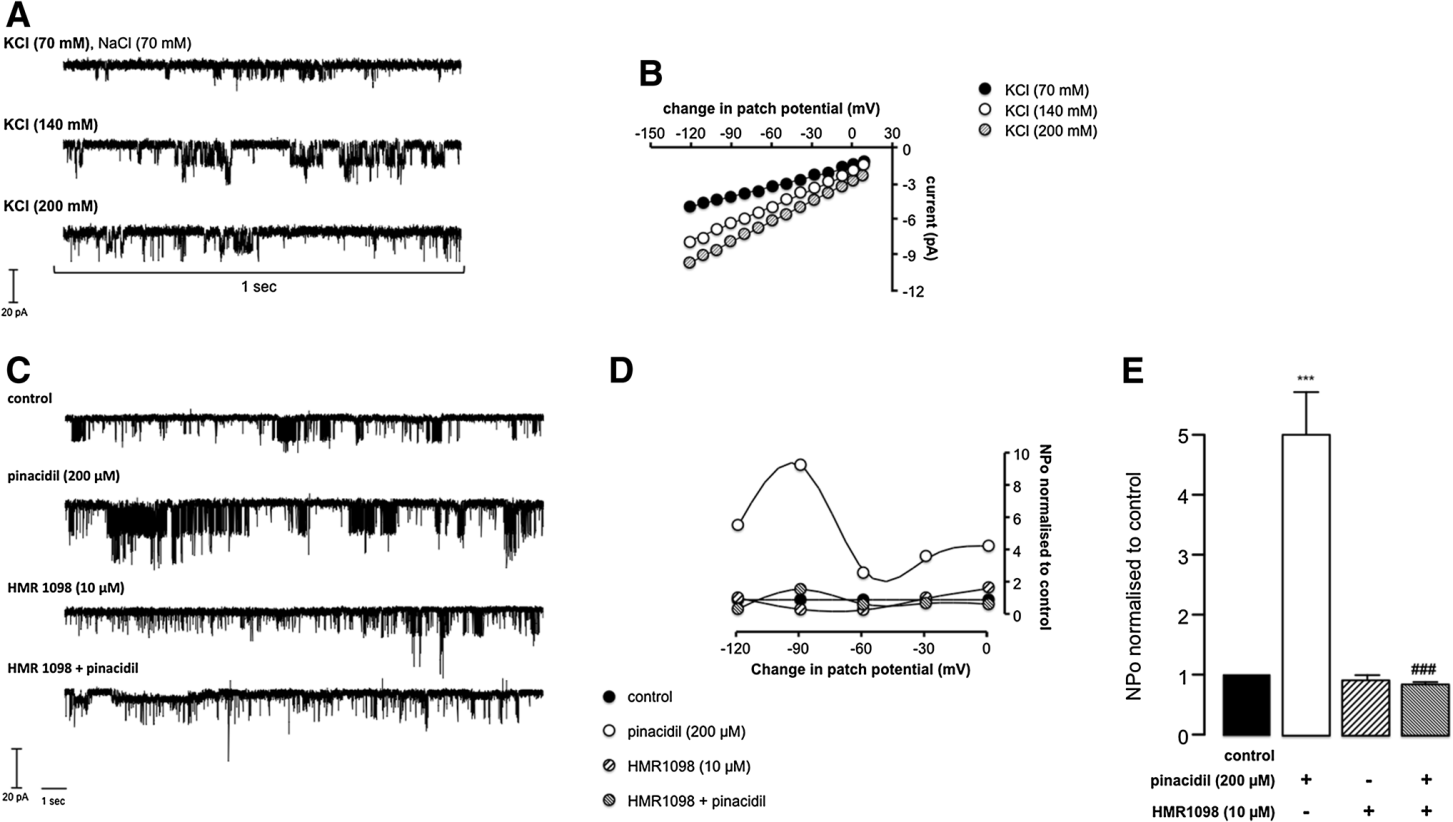
Representative sK_ATP_ recordings (**a** and **c**), current–voltage plots (**b**), relationship between open probability and patch potential change (**d**) and open probability histograms (**e**). Data are mean ± SEM. ***P < 0.001 versus control and ^###^ *P* < 0.001 versus pinacidil (**e**), one-way ANOVA with Newman-Keuls post hoc test

Differing sK_ATP_ openings, unitary currents and conductance states were observed in cell-attached patches for each patch pipette configuration (Fig. 3a, b). Changes in KCl concentration (70, 140 and 200 mM) and interpolation of current voltage relationships yielded unitary conductances of 44.03 ± 1.38, 54.77 ± 1.83, and 61.31 ± 1.87 pS. An increase in unitary conductance was seen when the concentration of KCl in the patch pipette was increased, and the changes observed were statistically significant (44.03 ± 1.38 versus 54.77 ± 1.83 pS, *P* < 0.01; 61.31 ± 1.87 versus 54.77 ± 1.83 pS, *P* < 0.05). The intermediary unitary conductances are probably indicative of a functional chimeric sK_ATP_ with likely co-assembled pore forming subunits of K_ir_6.1 and 6.2 coupled with SUR2A [12].

**Fig. 4:**
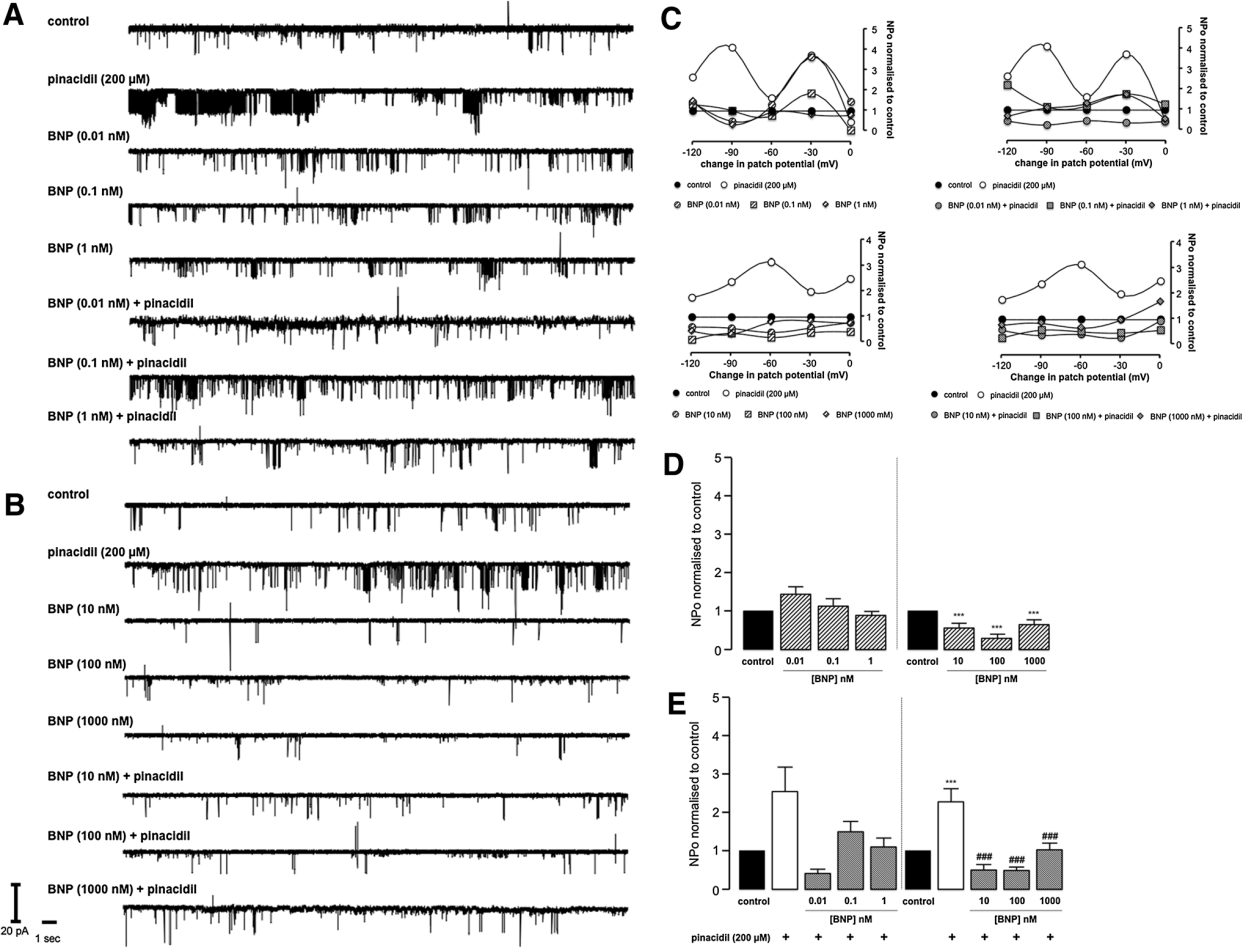
Representative sK_ATP_ recordings (**a** and **b**), the relationship between open probability and patch potential change (**c**) and open probability histograms (**d** and **e**). Data are mean ± SEM. ***P < 0.001 versus control (**d**), one-way ANOVA with Dunnett post hoc test; ***P < 0.001 versus control and ^###^ *P* < 0.001 versus pinacidil (**e**), one-way ANOVA with Newman-Keuls post hoc test

**Fig. 5:**
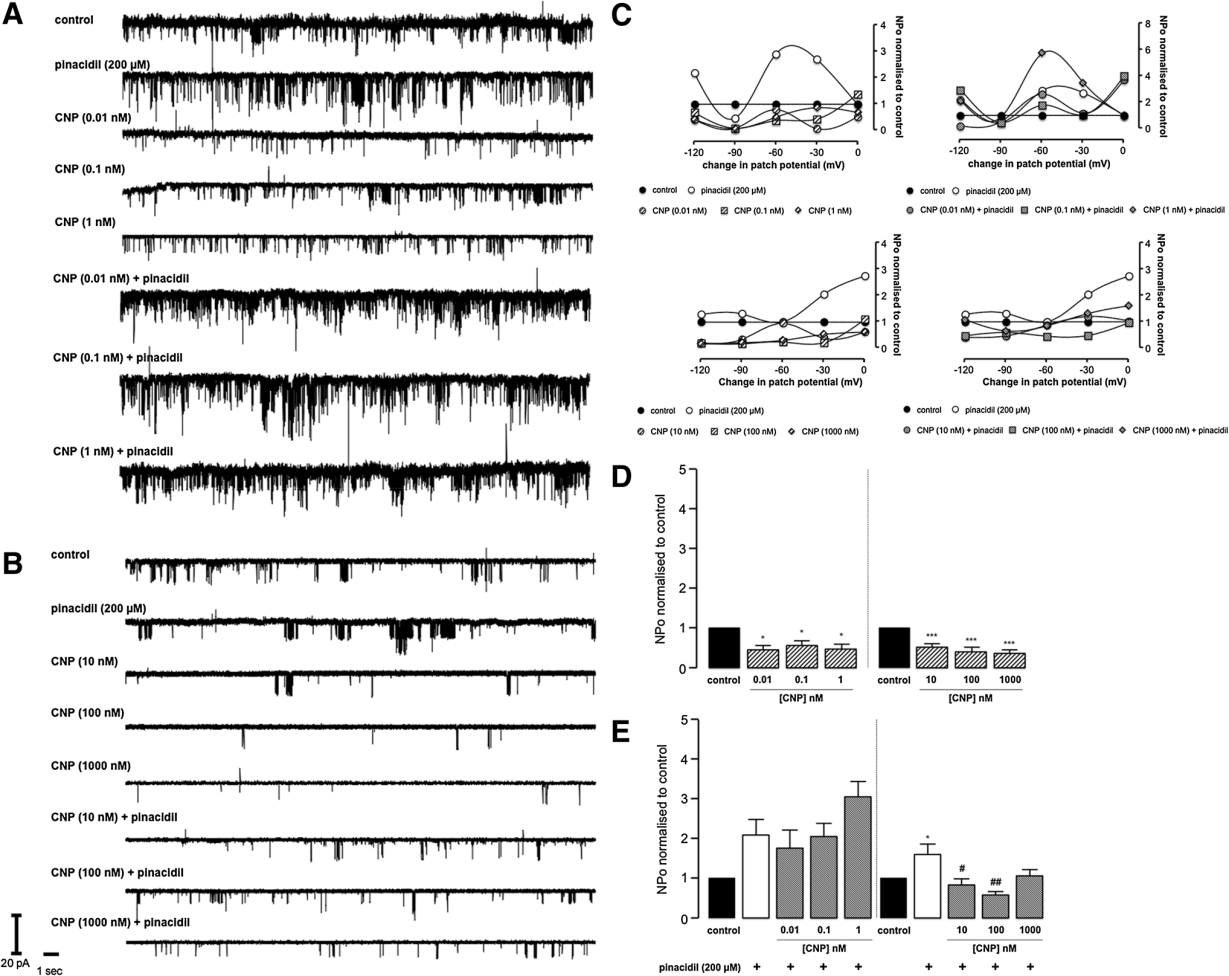
Representative sK_ATP_ recordings (**a** and **b**), the relationship between open probability and patch potential change (**c**) and open probability histograms (**d** and **e**). Data are mean ± SEM. *P < 0.05 and ***P < 0.001 versus control (**d**), one-way ANOVA with Dunnett post hoc test; *P < 0.01 versus control, ^#^ *P* < 0.05 and ^##^ *P* < 0.01 versus pinacidil (**e**), one-way ANOVA with Newman-Keuls post hoc test

**Fig. 6:**
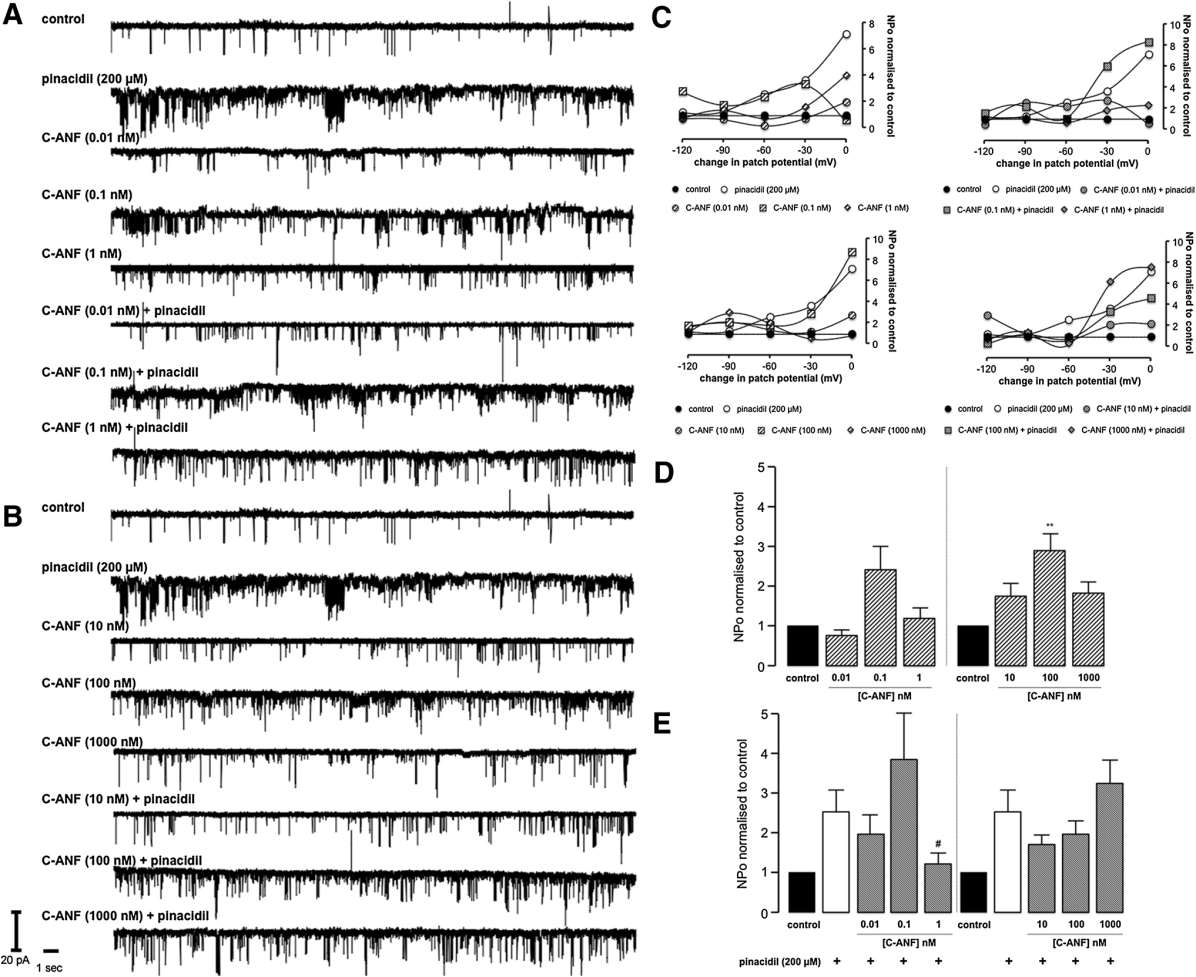
Representative sK_ATP_ recordings (**a** and **b**), the relationship between open probability and patch potential change (**c**) and open probability histograms (**d** and **e**). Data are mean ± SEM. **P < 0.01 versus control (**d**), one-way ANOVA with Dunnett post hoc test; ^#^ *P* < 0.05 versus pinacidil (**e**), one-way ANOVA with Newman-Keuls post hoc test

**Fig. 7:**
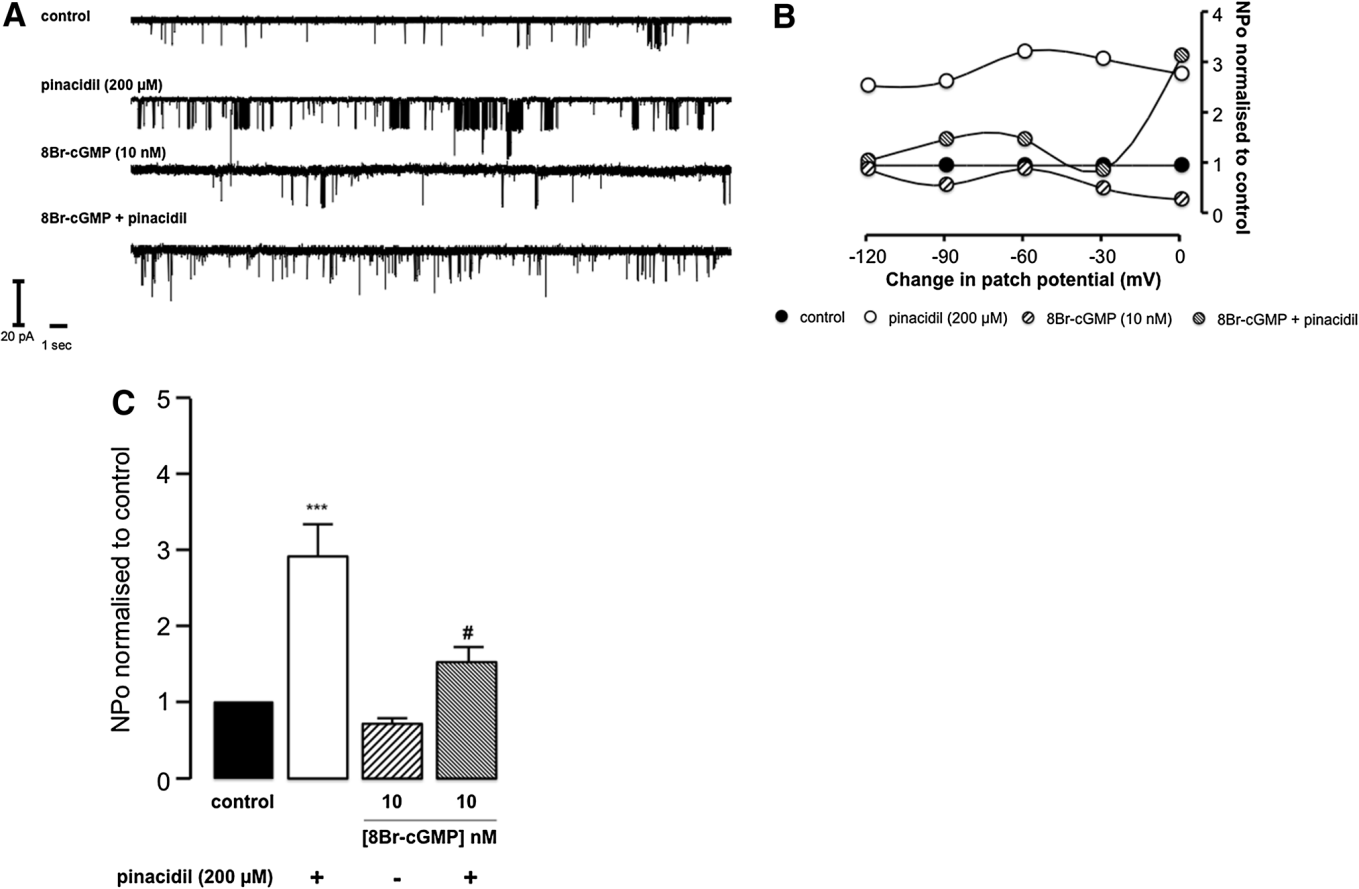
Representative sK_ATP_ recordings (**a**), the relationship between open probability and patch potential change (**b**) and open probability histograms (**c**). Data are mean ± SEM. ***P < 0.001 versus control and ^#^ *P* < 0.05 versus pinacidil (**c**), one-way ANOVA with Newman-Keuls post hoc test

In preliminary experiments, pinacidil was selected as the most consistently effective K_ATP_ opener. Bath application of pinacidil 200 μM had a marked effect on channel activity highlighted by a 5.2-fold increase in channel NPo compared to control (Fig. 3c–e; *P* < 0.001). In our hands, there was no relationship between NPo and patch potential change as illustrated in Figs. 3d, 4c, 5c, 6c and 7b. There was no significant difference in sK_ATP_ unitary conductance with pinacidil (*P* > 0.05; see Table 1). The selective sK_ATP_ inhibitor HMR1098 10 μM had no effect on basal channel activity and NPo, *P* > 0.05, but effectively reduced pinacidil-induced sK_ATP_ openings and NPo (Fig. 3c–e) to basal levels (83 % reduction; 5.23 ± 1.20 versus 0.89 ± 0.18; *P* < 0.001). There was no significant difference in sK_ATP_ unitary conductance with HMR1098 (*P* > 0.05; see Table 1).

**Table 1:** Current voltage data, group number in round brackets

Treatment	Number of cells	Unitary conductance (pS)	Reversal potential (mV)	NPo
1	4	44.03 ± 1.38	52.69 ± 1.86	–
2	5	54.77 ± 1.83	52.91 ± 0.92	–
3	4	61.31 ± 1.87	55.06 ± 2.25	–
4	12	57.08 ± 3.10	46.71 ± 5.44	1.00
5	7	61.93 ± 5.37	52.42 ± 19.10	5.23 ± 1.20
6	8	60.94 ± 5.67	46.46 ± 8.97	0.92 ± 0.26
7	6	53.42 ± 6.15	82.24 ± 26.28	0.89 ± 0.18
8	8	59.61 ± 4.94	2.90 ± 11.47	1.00
9	10	56.78 ± 2.34	22.19 ± 7.46	2.55 ± 0.88
10	5	56.62 ± 4.13	29.30 ± 11.71	1.44 ± 0.28
11	3	57.93 ± 2.89	31.52 ± 19.94	1.14 ± 0.28
12	5	56.04 ± 4.56	7.96 ± 13.97	0.85 ± 0.16
13	3	45.93 ± 11.78	29.06 ± 13.75	0.41 ± 0.05
14	5	65.08 ± 6.40	7.19 ± 4.23	1.50 ± 0.30
15	3	52.70 ± 6.70	43.06 ± 5.35	1.10 ± 0.29
16	35	52.91 ± 1.66	26.11 ± 6.05	1.00
17	41	59.61 ± 1.96	34.98 ± 4.61	2.28 ± 0.28
18	9	53.83 ± 2.03	22.29 ± 12.70	0.56 ± 0.09
19	8	62.05 ± 6.84	32.84 ± 12.66	0.29 ± 0.06
20	14	56.69 ± 4.47	50.97 ± 7.75	0.65 ± 0.28
21	9	66.41 ± 5.32	37.41 ± 8.90	0.50 ± 0.08
22	8	55.75 ± 2.52	50.73 ± 4.75	0.49 ± 0.28
23	10	51.48 ± 2.92	50.55 ± 5.80	1.03 ± 0.13
24	5	62.92 ± 4.08	21.11 ± 14.10	1.00
25	4	69.8 ± 4.87	7.73 ± 13.09	2.08 ± 0.50
26	3	58.13 ± 12.26	-1.96 ± 30.88	0.45 ± 0.16
27	3	61.57 ± 5.89	30.93 ± 23.62	0.57 ± 0.20
28	3	67.47 ± 1.69	33.42 ± 23.41	0.47 ± 0.14
29	4	45.93 ± 11.78	29.06 ± 13.75	1.76 ± 0.62
30	3	65.08 ± 6.40	7.19 ± 4.23	2.05 ± 0.54
31	4	52.70 ± 4.70	43.06 ± 5.35	3.05 ± 0.84
32	29	52.07 ± 2.03	25.61 ± 7.01	1.00
33	34	58.53 ± 2.33	29.91 ± 4.90	1.61 ± 0.20
34	10	53.37 ± 3.63	35.10 ± 12.04	0.52 ± 0.28
35	9	47.51 ± 3.16	45.25 ± 10.70	0.40 ± 0.13
36	16	56.73 ± 3.30	33.43 ± 8.64	0.36 ± 0.06
37	8	51.81 ± 3.67	20.65 ± 10.72	0.83 ± 0.28
38	8	45.31 ± 5.37	29.18 ± 9.20	0.58 ± 0.10
39	8	59.23 ± 4.21	32.22 ± 10.57	1.06 ± 0.17
40	7	65.03 ± 8.39	20.96 ± 13.27	1.00
41	11	62.03 ± 4.72	4.63 ± 9.50	2.54 ± 0.60
42	6	54.00 ± 6.06	-3.90 ± 13.61	0.76 ± 0.16
43	3	58.90 ± 14.08	9.50 ± 16.35	2.43 ± 0.84
44	5	56.30 ± 2.48	-3.15 ± 13.87	1.19 ± 0.30
45	7	49.00 ± 4.31	14.12 ± 8.52	1.97 ± 0.55
46	4	63.55 ± 6.81	0.54 ± 27.84	3.85 ± 1.13
47	7	53.47 ± 7.80	0.30 ± 14.37	1.22 ± 0.29
48	5	46.66 ± 3.11	7.07 ± 12.65	1.75 ± 0.40
49	4	64.65 ± 5.28	5.13 ± 11.65	2.90 ± 0.76
50	5	55.60 ± 4.23	15.95 ± 15.72	1.83 ± 0.50
51	4	63.10 ± 5.64	3.78 ± 7.27	1.71 ± 0.38
52	4	60.98 ± 11.80	29.33 ± 7.09	2.00 ± 0.53
53	6	53.22 ± 3.48	30.34 ± 16.44	3.25 ± 0.88
54	18	52.89 ± 2.55	13.26 ± 7.51	1.00
55	12	57.28 ± 5.52	11.47 ± 6.65	2.92 ± 0.60
56	10	55.78 ± 3.95	33.65 ± 8.67	0.72 ± 0.11
57	14	47.74 ± 3.05	31.78 ± 12.79	1.53 ± 0.32

### The effect of BNP and CNP on sK_ATP_ opening

Neither BNP nor CNP caused an appreciable increase in sK_ATP_ activity (Figs. 4a–e, 5a–e); in fact, bath application of either peptide resulted in a decrease in channel activity. BNP (≥10 nM) and CNP (≥0.01 nM) caused a significant decrease in sK_ATP_ NPo compared to control (Figs. 4a–e, 5a–e). For BNP and CNP, this decrease was concentration dependent (Figs. 4d, 5d). BNP at low concentrations (≤1 nM) had no effect on sK_ATP_ current and NPo (Fig. 4d). When either BNP (≥10 nM) or CNP (≥10 nM) was applied with pinacidil, the effects of the sK_ATP_ opener were completely abolished, highlighted by a marked reduction in channel NPo down to or below basal level. This significant effect was seen with BNP at all concentrations ≥10 nM [Fig. 4e: 2.28 ± 0.28 versus 0.50 ± 0.08 (10 nM), 0.49 ± 0.07 (100 nM), 1.03 ± 0.13 (1,000 nM); *P* < 0.001]. CNP at two out of three concentrations had similar effects [Fig. 5e: 1.61 ± 0.20 versus 0.83 ± 0.15 (10 nM), 0.58 ± 0.10 (100 nM); *P* < 0.05 and *P* < 0.01, respectively]. BNP (≤1 nM) did reduce pinacidil-stimulated sK_ATP_ currents but these effects did not reach significance (Fig. 4e; *P* > 0.05); furthermore, CNP applied at low concentrations (≤1 nM) was incapable of inhibiting pinacidil-stimulated sK_ATP_ currents (Fig. 5e; *P* > 0.05). These effects were not voltage dependent (Figs. 4c, 5c), and all treatments (see Table 1) had no significant effect on single channel unitary conductance compared to control.

### The effect of C-ANF on sK_ATP_ activity

The NPR-C agonist C-ANF had interesting effects on sK_ATP_ activity. C-ANF (0.01 and 1 nM) had a negligible sK_ATP_ NPo; however, a 2.4-fold increase in NPo was seen with C-ANF 0.1 nM compared to control; however, this was not significant (Fig. 6d; *P* > 0.05). C-ANF (≥10 nM) augmented sK_ATP_ activity although a significant effect on sK_ATP_ NPo was only seen with C-ANF 100 nM (Fig. 6d; *P* < 0.01); nevertheless, C-ANF 10 and 1,000 nM caused an appreciable increase in sK_ATP_ activity (Fig. 6d). C-ANF 1 nM caused a significant blunting of pinacidil stimulated sK_ATP_ currents (2.54 ± 0.6 versus 1.22 ± 0.29 (1 nM); *P* < 0.05); however, this effect was not seen at all the other concentrations tested (Fig. 6e). The effects exhibited by C-ANF at all other concentrations were not statistically significant, although modest dampening of pinacidil stimulated sK_ATP_ activity is still evident at some concentrations (Fig. 6e; *P* > 0.05). There was no significant effect on single channel unitary conductance compared to control, and effects on NPo were not voltage dependent (*P* > 0.05; Fig. 6c and Table 1).

### Cyclic GMP effect on sK_ATP_ activity

Unlike BNP and CNP, application of 8Br-cGMP (10 nM) did not cause a significant decrease in basal sK_ATP_ activity compared to control (*P* > 0.05). However, the compound attenuated sK_ATP_ responses to pinacidil when given simultaneously, causing a reduction in NPo (Fig. 7c). This 50 % relative reduction in NPo was significant (2.92 ± 0.60 versus 1.53 ± 0.32; *P* < 0.05). There was no appreciable effect on single channel conductance compared to control, and effects on NPo were not voltage dependent (*P* > 0.05; Fig. 7b and Table 1).

## Discussion

### Principal findings

Our data confirm the existence and expression of all K_ATP_ subunit genes and proteins in ventricular cardiomyocytes using RT-PCR and Western blotting and patch clamping, revealing a functional sK_ATP_ with biophysical and pharmacological properties consistent with that reported in the literature [46]. Our data also demonstrate a novel natriuretic peptide receptor mechanism of sK_ATP_ regulation in the cardiomyocyte under normoxic conditions. BNP (≤1 nM) had no effect on basal sK_ATP_ current but at high concentrations (≥10 nM) inhibited the ion channel, reducing NPo. BNP suppressed pinacidil-stimulated sK_ATP_ currents at all concentrations with the most marked effect seen at concentrations ≥10 nM. CNP (≥0.01 nM) suppressed basal sK_ATP_ openings, but only displayed this inhibitory action in presence of pinacidil at concentrations ≥10 nM. C-ANF (≥10 nM) had a marked stimulatory effect on basal sK_ATP_; however, the effects of C-ANF at low concentrations (≤1 nM) are inconsistent. C-ANF had a negligible blunting effect on pinacidil-stimulated sK_ATP_ currents at all concentrations except at 1 nM. The action of BNP and CNP could potentially be associated with their ability to elevate intracellular concentrations of the second messenger cGMP, as it was demonstrated that the analog 8Br-cGMP was capable of dampening the pinacidil-activated sK_ATP_ current. As complete speculation, the stimulatory action of C-ANF at high concentrations (≥10 nM) could be associated with NPR-C mediated activation of PKC and subsequent sK_ATP_ opening [2, 24, 37].

### Biomolecular, biophysical and pharmacological characterization of rat ventricular K_ATP_

In this study, RT-PCR and Western blotting demonstrated the presence of K_ir_6.1, K_ir_6.2, SUR1 and SUR2 genes and proteins in rat left ventricular cardiomyocytes (Figs. 1, 2). Using immunofluorescence microscopy, Morrissey and colleagues confirmed the expression of all four K_ATP_ subunit proteins in rat ventricular cardiomyocytes, observing the co-localization of K_ir_6.2 and SUR2 subunits in the sarcolemma and transverse t-tubules [27]. Additionally, they found that K_ir_6.1 and SUR1 expression was particularly strong at the sarcolemmal surface [27]. Concerning the expression of SUR1 in our study, a strong band was detected at 70 kDa rather than the predicted 174 kDa. It is not known if the 70 kDa band was unmasked due to non-specific binding of the primary antibody, or if a SUR1 short form splice variant was detected. Splice variants of SUR1 have already been described in the heart [14, 18, 32]; however, their biological significance requires further elucidation.

The single channel unitary conductance of sK_ATP_ in symmetrical K^+^ (140 mM) conditions was between 50 and 60 pS (see Table 1). A sK_ATP_ was described in adult rat ventricular cardiomyocytes with a unitary conductance of 57.2 pS [46]. However, a considerable body of evidence has reported the unitary conductance of K_ATP_ in guinea pig [29], human [3], mouse [4], rabbit [29] and rat [48] ventricular cardiomyocytes to be between 70 and 80 pS under similar experimental conditions. This disparity in channel conductance reported in this study compared to that historically reported can be explained by the role of K_ir_6.X subunits in dictating K_ATP_ conductance [34]. K_ir_6.1 and K_ir_6.2 are highly homologous proteins that form a functional K^+^ channel when coupled to a SUR (1:1 tetrameric stoichiometry), with remarkably different unitary conductance. Under symmetrical K^+^ conditions, K_ir_6.1/SURX and K_ir_6.2/SURX have a divergent unitary conductance approximating 35 and 80 pS, respectively [34]. Chimeras of K_ATP_ have been described as exhibiting an intermediary unitary conductance between 55 and 65 pS [4, 12, 25, 42]. In two independent studies, the unitary conductance of K_ATP_ in cardiac cells isolated from mouse and rabbit purkinje fibers was demonstrated to be 57.1 pS [4] and 60.1 pS [25], respectively. Bao and colleagues proposed that the channel observed in their inside-out patch clamp experiments was a chimeric K_ATP_ [4]. Intriguingly following each attempted excision of the patch, channel activity completely disappeared, and according to Bao and co-workers, this could be indicative or a characteristic of a heteromeric K_ATP_ [4]. Morrissey and co-workers put forward the notion that a reassessment of the molecular composition of K_ATP_ in ventricular myocytes is needed, after elegantly showing strong sarcolemmal expression of K_ir_6.1 and SUR1 subunits by means of immunofluorescence [27]. In light of all the evidence, it is possible that a heteromeric K_ATP_ is present in the cardiac sarcolemma that presumably comprised two pore forming subunits of K_ir_6.1 and 6.2 coupled with SUR2A. This could explain why in our hands, a sarcolemmal K^+^ channel with features associated with K_ATP_ with a unitary conductance 50–60 pS was evident in our cell-attached patches.

In cell-attached patches, sK_ATP_ activity was markedly upregulated by the K_ATP_ opener pinacidil (Fig. 3c–e), an effect not seen with diazoxide (data not shown). This finding was not surprising because K_ATP_ with SUR1 (atrium) [14] and SUR2B (smooth muscle) [49] is highly sensitive to diazoxide, but not the SUR2A form (ventricle) [1]. Typically, pinacidil 200 μM increased sK_ATP_ NPo several fold above basal, an effect that was completely abolished by the selective inhibitor of the membrane form of K_ATP_ HMR1098 10 μM (Fig. 3d, e). HMR1098 did not reduce basal K_ATP_ openings (Fig. 3d, e). HMR1098 at concentrations ≥100 μM would be sufficient to reduce basal K_ATP_ opening [50]. These data provide pharmacological evidence that the K^+^ channel observed in cell-attached patches was sK_ATP_.

### Natriuretic peptide receptor modulation of rat ventricular K_ATP_

Application of both BNP (≥10 nM) and CNP (≥0.01 nM) caused a marked and consistent depression of basal sK_ATP_ activity and NPo (Figs. 4, 5), contrary to our thinking that naturally occurring natriuretic peptides elicit/upregulate sK_ATP_ opening. The rationale behind our hypothesis that natriuretic peptides promote K_ATP_ opening was based on several studies in the setting of cardioprotection, showing that natriuretic peptide-induced limitation of infarct size involves K_ATP_ opening [5, 13, 47]. Thus, this study initially set out to investigate such a possibility by means of patch clamping, in particular the cell-attached configuration to maintain the intactness of intracellular signaling mechanisms, namely the natriuretic peptide receptor (NPR-A, NPR-B)/cGMP/protein kinase G (PKG) signaling cascade. The fascinating findings with both BNP and CNP on basal sK_ATP_ activity, together with their inhibitory effects on pinacidil evoked sK_ATP_ currents (Figs. 4, 5), appear to illustrate a novel mechanism of NPR-A and NPR-B regulation of sK_ATP_ in the heart. It is well known that natriuretic peptides play key roles in the cardiovascular adaptation to both acute and chronic pathological insult. The complexity of their fundamental roles as key mediators in multiple body systems, beyond the regulation of blood volume, is well documented, and it appears that the regulation of sK_ATP_ in the myocardium is an extension of this axis. Saegusa and colleagues demonstrated that ANP secretion from mechanically stretched mouse isolated atria was markedly enhanced in preparations taken from K_ir_6.2 deficient mice compared to wild type [38]. Speculatively, they suggested that sK_ATP_ could play a compensatory role in protecting the heart under pathological conditions. However, under physiological conditions, it could control stretch-induced ANP secretion via a negative feedback loop [38]. In a previous study, the sulphonylurea receptor ligand diazoxide, a K_ATP_ opener, was shown to inhibit stretch-induced ANP release in atrial cardiomyocytes [23], thus supporting the findings of Saegusa and colleagues [38].

BNP and CNP are agonists for different receptor-linked pGCs, namely NPR-A and NPR-B, respectively, and that both are capable of generating the second messenger cGMP. The fact that both BNP and ANP bind to the same receptor with the former having comparably lower affinity raises the possibility that the negative modulatory effects seen with BNP on K_ATP_ function can be recapitulated by ANP. Indeed Ropero and colleagues showed that ANP 1 nM dampened K_ATP_ activity in cell-attached patches from mouse pancreatic beta cells, illustrated by a 50 % reduction in NPo compared to no-treatment control [36]. The result obtained in Ropero’s study [36] is consistent with our findings that BNP and CNP are capable of inhibiting sK_ATP_ activity in rat ventricular cardiomyocytes.

The natriuretic peptides including BNP and CNP have a high affinity for the clearance receptor NPR-C [37]. Several sources of evidence suggest that some of the biological effects produced by natriuretic peptides are mediated through NPR-C, with evidence supporting a role for NPR-C in the hyperpolarization of vascular smooth muscle and endothelium [10, 44], and its role in CNP regulation of coronary blood flow and cardioprotection [22]. We sought to examine the role of NPR-C in natriuretic peptide regulation of sK_ATP_ using the NPR-C agonist C-ANF. Interestingly, C-ANF at concentrations ≥10 nM stimulated sK_ATP_ currents in our patch clamp experiments (Fig. 6). However, inhibition of pinacidil stimulated K_ATP_ currents was only observed with C-ANF 1 nM. These observations suggest that BNP and CNP do not elicit sK_ATP_ inhibition via NPR-C agonism.

### cGMP as a modulator of K_ATP_

**Fig. 8:**
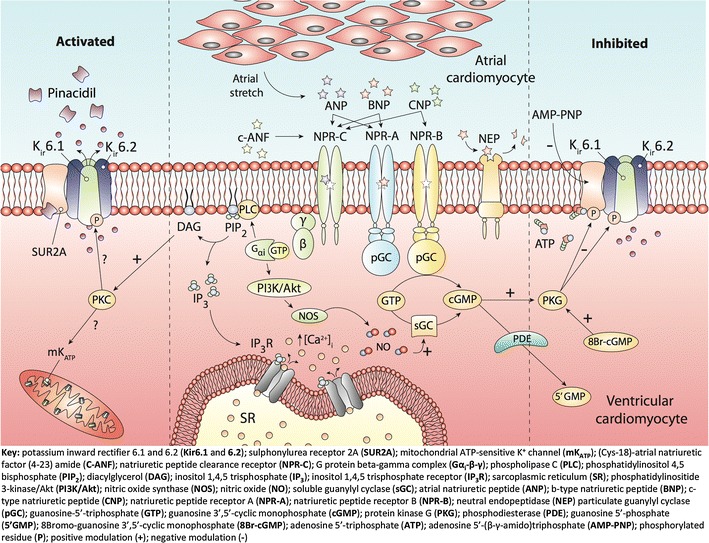
ANP/BNP and CNP bind cell surface receptors called NPR-A and NPR-B, respectively, which have an intracellular catalytic domain with guanylyl cyclase activity. NPR-A and NPR-B agonism leads to the generation of cGMP and activation of PKG. PKG phosphorylates serine/threonine residues in sK_ATP_ causing inhibition. The effect of NPR-A and NPR-B agonism on sK_ATP_ activity is mimicked by the cGMP analog 8Br-cGMP. C-ANF binds a distinct receptor devoid of a guanylyl cyclase domain called NPR-C and through a proposed Gα_i_-PLC-PIP_2_-DAG mechanism, activates PKC. PKC phosphorylates serine/threonine residues in sK_ATP_ leading to channel opening and an increase in sK_ATP_ activity. The PI3K/Akt/NOS and NO/sGC/cGMP signaling pathways have been proposed to interplay with the natriuretic pathway, augmenting natriuretic peptide generated pools of cGMP. These pools could potentially be responsible for facilitating sK_ATP_ inhibition

The cGMP analog 8Br-cGMP 10 nM had no appreciable effect on sK_ATP_ openings under normoxic conditions, with no reduction in sK_ATP_ NPo compared to control, however, caused 50 % inhibition of pinacidil stimulated K_ATP_ openings (Fig. 7). Taking into consideration the results obtained with 8Br-cGMP, BNP (≥10 nM) and CNP (≥0.01 nM), the unexpected and novel inhibitory action of the natriuretic peptides on cardiac K_ATP_ activity may be at least partially associated with their ability to augment intracellular cGMP concentrations. However, a recent study by Chai and co-workers found that 8Br-cGMP 500 μM caused a threefold increase in K_ATP_ NPo in cell-attached patches from rabbit ventricular cardiomyocytes, although the representative recordings are somewhat unconvincing [9]. Furthermore, the concentration of cGMP used in this study is excessive, and massively in excess of intracellular cGMP concentration [16]. Interestingly, they found that the cell-permeable cGMP-phosphodiesterase inhibitor zaprinast (0.05–50 μM) increased K_ATP_ NPo in a concentration-dependent manner up to 12-fold above baseline, an effect that was completely blunted by addition of the PKG inhibitor KT5823 1 μM [9]. Their data show that cGMP-induced increase in sK_ATP_ activity in rabbit ventricular myocytes is in part PKG dependent. An earlier study by Han and colleagues examined the effect on NO on K_ATP_ activity in rabbit ventricular cardiomyocytes [20]. In cell-attached patches stimulated with pinacidil 50 μM, cumulative application of the NO-donors SNP or SNAP (0.1–1,000 μM) resulted in a concentration-dependent increase in K_ATP_ Po, an effect that was abolished by the K_ATP_ inhibitor glibenclamide 30 μM [20]. Furthermore, the potentiating effects of both NO-donors on pinacidil-induced K_ATP_ openings were abrogated by two structurally different PKG inhibitors Rp-8-Br-PET-cGMPS 10 μM and Rp-pCPT-cGMP 100 μM [20]. Similar findings were presented in a latter study, alluding to PKG activation as the key mechanism by which cGMP and NO-donors activate K_ATP_ [19] Fig. 8.

Taking all these findings into consideration, it appears that natriuretic peptides and NO have opposing effects on K_ATP_ activity cardiomyocytes, consistent with the differential effects observed with both autacoids despite generating the same second messenger [7, 8, 41]. Determining cGMP concentration following BNP and CNP administration in our pinacidil-activated preparation would give an interesting insight into the relationship between natriuretic peptide signaling and K_ATP_ activity. However, limitations remain using primary cultures of adult rat ventricular heart tissue that have prevented us from attempting such measurements relating to sufficient tissue, sensitivity to calcium during the isolation process and phenotypic stability [11].

### Pathophysiological implications

The work described here has been undertaken in cardiomyocytes examined under standard electrophysiological conditions (normoxia). The technical limitations of the approach preclude the examination of natriuretic peptide effects on K_ATP_ under conditions of hypoxia or oxidative stress relevant to cardiac pathologies such as ischemia–reperfusion or ischemic cardiomyopathy. K_ATP_ is implicated in arrhythmia genesis [15], and mutations in genes coding for K_ir_6.2 (KCNJ11) and SUR2 (ABCC9) are linked to left ventricular hypertrophy and dilated cardiomyopathy in humans [30]. It will be relevant to attempt to model these in future studies. Although the concentrations of BNP and CNP employed in some experiments are many times higher than picomolar physiological plasma concentrations [35], they are very relevant to the interstitial concentrations in ventricular myocardium, especially in pathological states [35]. In conditions characterized by left ventricular dysfunction, such as chronic heart failure, release of stored BNP is observed (and there is some evidence to suggest CNP also), resulting in myocardial concentrations in the nanomolar region [35].

## Conclusion

In conclusion, we have shown that BNP and CNP inhibit sK_ATP_ in rat ventricular cardiomyocytes and we believe this to be a novel NPR-A and NPR-B mechanism of K_ATP_ regulation in the heart, at least under physiological conditions. Examination of this regulatory mechanism in cardiomyocytes under conditions of oxygen deprivation and whether there are fundamental changes in natriuretic peptide regulation of K_ATP_ is important and warrants future investigation.

## Electronic supplementary material

Below is the link to the electronic supplementary material.

Supplementary material 1 (DOCX 73 kb)
