# Supplementary material for: Natriuretic peptides modulate ATP-sensitive K+ channels in rat ventricular cardiomyocytes
Source: Basic Res Cardiol. 2014 Jan 30;109(2):402. doi: 10.1007/s00395-014-0402-4 (PMC3951884; doi:10.1007/s00395-014-0402-4)
Supplement: Supplementary file 1 — Supplementary material 1 (DOCX 73 kb) [file 395_2014_402_MOESM1_ESM.docx]

**Natriuretic peptides inhibit ATP-sensitive K^+^ channels in rat ventricular cardiomyocytes**

**Basic Reasearch in Cardiology**

Dwaine S. Burley, PhD; Charles D. Cox, PhD; Jin Zhang, PhD;

Kenneth T. Wann, PhD; Gary F. Baxter, PhD, DSc

Cardiff School of Pharmacy and Pharmaceutical Sciences, Cardiff University, UK

**Address for correspondence:**

Dwaine S. Burley PhD

School of Pharmacy and Pharmaceutical Sciences

Cardiff University

King Edward VII Avenue

Cardiff

CF10 3NB

United Kingdom

Telephone: +44 (0)29 2087 6446

Fax: +44 (0)29 2087 4149

Email: [burleyds@cardiff.ac.uk](mailto:baxtergf@cardiff.ac.uk)

**SUPPLEMENTAL MATERIAL**

**Supplementary methods**

**1. Animals**

Forty-four male Sprague-Dawley rats (270-350g) obtained from Harlan Laboratories (Bicester, Oxford) were used in this study. Rats were kept in an institutional animal house and maintained in a constant temperature, humidity and ventilation controlled environment. They were subjected to light/dark cycles, 12 hours for each period, and allowed to acclimatise for a minimum of 7 days prior use. During this and consecutive housing periods, rats were fed on chow containing 4% fat and 18% protein (Harlan-Teklad diet) and had free access to water at all times. The care and use of animals were in accordance with the UK Home Office guidelines on the Animals Scientific Procedures Act 1986 published by Her Majesty’s Stationary Office.

**2. Adult rat cardiomyocyte isolation**

Rats were anaesthetised with sodium pentobarbital (230 mg/kg) by intra-peritoneal injection. Following thoracotomy, hearts were rapidly excised and immersed in ice-cold Krebs-Henseleit buffer (KHB) prior to perfusion. The composition of KHB in mM was: 118 NaCl, 25 NaCO_3_, 11 D-glucose, 4.7 KCl, 1.22 MgSO_4_, 1.21 KH_2_PO_4_, 1.25 CaCl_2_. Hearts were then Langendorff perfused with aerated (95% O_2_/ 5% CO_2_) KHB pH 7.4 at constant flow (12 mL/min) and maintained at 37-38°C. Following a 5 min perfusion with KHB, hearts were perfused with low Ca^2+^-HEPES buffer pH 7.4 for 5 min, followed by a 30 min perfusion with enzyme buffer pH 7.4 containing Liberase Blendzyme DL (0.125 mg/mL). The composition of both buffers in mM is identical apart from the concentration of Ca^2+^ and the addition of nitrilotriacetic acid (NTA) and 2,3-butanedione monoxime (BDM) to the low Ca^2+^ and enzyme buffers respectively: 120 NaCl, 20 D-glucose, 5.4 KCl, 5 MgSO_4_, 5 pyruvate, 20 taurine, 10 HEPES; 5 NTA and 0.014 CaCl_2_ (low Ca^2+^); 10 BDM and 0.2 CaCl_2_ (enzyme). After completion of the perfusion protocol, atria and right ventricle were excised and discarded. The remaining left ventricle, left apex and septum were cut into small pieces and agitated in enzyme buffer on a hot plate stirrer at 400 rpm at 37°C until completely digested. The suspension consisting of rod shaped cardiomyocytes was centrifuged at 1000 rpm for 1 min. After gently discarding the supernatant, the remaining pellet was resuspended in medium 199 with Earle’s salts and HEPES 25 mM (Invitrogen, UK) supplemented with the following in mM unless otherwise stated: 5 creatine, 5 taurine, 2 L-carnitine, 2.5 pyruvate, 100 nM insulin, 50 IU Penicillin and 50 μg streptomycin (Sigma-Aldrich, UK). Cardiomyocytes were seeded at a density of 20,000 rods/well on extracellular matrix gel coated (8 µg) plastic coverslips, and cultured overnight under normal CO_2_ incubator conditions at 37°C, prior to treatments and patch clamping.

**3. PCR**

**3.1 RNA extraction**

Following the enzymatic tissue digestion protocol as outlined in section 2, cardiomyocyte rich pellet(s) were resuspended in 1.25 mL TRIzol reagent (Invitrogen, UK) and 250 μL of chloroform. Following gentile agitation, microcentrifuge tubes containing samples were spun at 14000 rpm, for 10 min at room temperature. Two phases were observed, the pink coloured chloroform phase containing organic contents, and a colourless aqueous phase. The colourless aqueous solution containing RNA was aspirated and transferred to 1.5 mL tubes. Isopropyl alcohol was added to each tube to promote RNA precipitation; the content of each tube was allowed to incubate for 10 min at room temperature prior to centrifugation using the same parameters mentioned previously. After centrifugation the supernatant in each tube was carefully removed in order not to damage the RNA pellet, each pellet was then agitated in 500 μL of ethanol (75% v/v), vortexed and centrifuged at 14000 rpm for 5 min. Following this, supernatants were aspirated and tubes were left open to air to allow evaporation of any remaining ethanol. Finally 25 μL of nuclease free H_2_O was added to each tube, tubes (x2) were then stored at -80°C overnight.

In order to remove the remaining DNA in the RNA sample, samples were treated with DNAse. In brief, 50 μL of RNA sample, 5 μL of 10x DNAse I buffer and 1 μL of rDNAse I were mixed and incubated in a thermal cycler at 37°C for 30 min. Following this, 5 μL of DNAse inactivation buffer was added to the mixture, and left to incubate for 2 min at room temperature whilst being subjected to vortex after each min. The mixture was then centrifuged at 10000 rpm for 2 min at room temperature, the resulting RNA rich supernatant was transferred to 200 μL tubes for storage prior to RNA quantification, which was done using spectrophotometry.

**3.2 Reverse transcription**

Reverse transcription (RT) was performed to prepare complementary DNA (cDNA) for the polymerase chain reaction (PCR). To test for DNA contamination in each RNA sample, a no-RT reaction was performed as a negative control.

**Table 1** RNA mixture

| Reagent | Amount added |
| --- | --- |
| RNA | 1 μg |
| Random primers (500 μg/μL) | 0.5 μg/μL |
| Nuclease free H_2_O | Up to 5 μL |

RNA mixtures were incubated at 70°C for 5 min then immediately chilled on ice. After this step, the following samples were prepared, as shown in table 2.

**Table 2** Reagents and reaction mixture for RT

| Reagent | RT-PCR (sample) | no-RT PCR (-ve control) |
| --- | --- | --- |
| 5× Reaction Buffer | 4 μL | 4 μL |
| MgCl_2_ (25 mM) | 2.4 μL | 2.4 μL |
| dNTPs (40 mM) | 1 μL | 1 μL |
| 0.5 μL RNAsin | 0.5 μL | 0.5 μL |
| Reverse Transcriptase | 1 μL | 0 μL |
| Nuclease free H_2_O | 6.1 μL (up to 15 μL) | 7.1 μL (up to 15 μL) |

Samples were incubated in a thermocycler (Perkin Elmer Catus) using the following program: 25°C x 5min, + 42°C x 60 min, + 70°C x 15 min. Both the RT and no-RT samples were stored at -20°C.

**3.3 Primers**

GAPDH (223 bp), K_ir_6.1 (KNCJ8, 227 bp), K_ir_6.2 (KNCJ11, 201 bp), SUR1 (ABCC8, 169 bp) and SUR2 (ABCC9, 228 bp) primers were obtained from Invitrogen (see table 3). Each primer was dissolved in Tris-EDTA buffer pH 8.0, to give a 100 μM stock. Stock solutions were then diluted 10-fold with nuclease free H_2_O, and stored at -20°C.

**Table 3** Forward and reverse primers; *Rattus Norvegicus*

| Primer name | Forward | Reverse |
| --- | --- | --- |
| Kir6.1 (KNCJ8) | 5’-TGAGTCTAGGACGCGTTGTG-3’ | 5’-TGCTCTCGGATGTTCTTGTG-3’ |
| Kir6.2 (KNCJ11) | 5’-CACTTTGTCTGGGGGACACT-3’ | 5’-TGGGAGGCTTTATTGACGAC-3’ |
| SUR1 (ABCC8) | 5’-CCCTCTACCAGCACACCACT-3’ | 5’-CAGTCAGCATGAGGCAGGTA-3’ |
| SUR2 (ABCC9) | 5’-AATCCTGTTCAGCGGCTCTA-3’ | 5’-CAACGCTGAAGTTCTCACCA-3’ |
| GAPDH | 5’-GGCATTGCTCTCAATGACAA-3’ | 5’-TGTGAGGGAGATGCTCAGTG-3’ |

**3.4 PCR**

The amplification of a house keeping gene cDNA was used as a positive control. GAPDH was selected as a positive control. The negative control was a non-amplified product; there was no reverse transcriptase. The following PCR mixtures were prepared, see table 4.

**Table 4** Reaction mixtures for PCR

| Reagents | +ve control | -ve control | Sample |
| --- | --- | --- | --- |
| Nuclease free H_2_O | 7.4 μL | 7.4 μL | 7.4 μL |
| Go Taq | 2.5 μL | 2.5 μL | 2.5 μL |
| MgCl_2_ (25 mM) | 0.75 μL | 0.75 μL | 0.75 μL |
| GAPDH primer forward | 0.5 μL | 0.5 μL | - |
| GAPDH primer reverse | 0.5 μL | 0.5 μL  **and** | - |
| Target primer forward | - | 0.5 μL | 0.5 μL |
| Target primer reverse | - | 0.5 μL | 0.5 μL |
| no-RT | - | 0.5 μL | - |
| cDNA | 0.5 μL | - | 0.5 μL |
| dNTP mix (10 mM) | 0.25 μL | 0.25 μL | 0.25 μL |
| Go Taq DNA polymerase  (5 U/μL) | 0.0625 μL | 0.0625 μL | 0.0625 μL |

Once all the relevant samples were prepared, tubes were placed in the PCR thermocycler and subjected to the following PCR amplification conditions: 95°C x 2 min [95°C x 30 sec + 55°C x 45 sec + 75°C x 1 min] x 30 + 72°C x 5 min + 4°C till the end***.*** PCR products and DNA ladder (hyperladder II 50-2000 bp) were mixed with Novel Juice (6x sample buffer) (genedirex, USA), a non-mutagenic nucleic acid stain, prior to separation by gel electrophoresis (1% agarose gel) at 100 mV for 6 hrs. DNA bands were visualized and photo acquired, following ultraviolet trans illumination of the agarose gel.

**4. Western blotting**

**4.1 Sample preparation**

As mentioned in section 3, following enzymatic tissue digestion, cardiomyocyte rich pellet(s) were resuspended and lysed in ice cold buffer containing the following in mM unless otherwise stated: 100 NaCl, 10 KCl, 3 MgCl_2_.6H_2_O, 0.25% v/v Triton X-100, 20 Tris-HCl pH 7.6, 10 EDTA pH 8.0, 5% v/v protease inhibitor cocktail (Sigma Aldrich, UK). All samples were lysed in microcentrifuge tubes. Cell lysate samples were subjected to 5 sec sonication then spun at 13000 rpm for 15 minute at 4°C. The resulting supernatants were aliquoted and diluted 1:2 with 2x Laemmli buffer, however a small volume of undiluted supernatant was saved for protein determination by BCA assay.

**4.2 Electrophoresis and detection**

Denatured protein samples were separated in 10% sds-polyacrylamide gels at 120 mV for 90 min. The following amounts of protein were loaded in order to help detect each K_ATP_ subunit: 80 μg for K_ir_6.1, 80 μg for K_ir_6.2, and 100 μg for SUR1 and SUR2. Proteins were then wet transferred on to methanol activated Hybond P PVDF (GE healthcare, UK) membranes in ice cooled transfer buffer at 100 mV for 60 min. Proteins were blocked in 5% milk TBS-tween for 3 hrs at room temperature in order to prevent non-specific binding of primary antibodies. Using the appropriate primary antibody, samples were then probed for K_ATP_ subunit proteins K_ir_6.1, K_ir_6.2, SUR1, SUR2 or the house keeping protein GAPDH overnight at 4°C: rabbit polyclonal to K_ir_6.1 (1:200); rabbit polyclonal to K_ir_6.2 (1:500); rabbit polyclonal to SUR1 (1:1000); rabbit polyclonal to SUR2 (1:1000); mouse polyclonal to GAPDH (1:50000). The following day, PVDF membranes were washed and incubated with the relevant secondary antibody diluted in 5% milk TBS-tween, for 60 min at room temperature: goat anti-rabbit HRP (1:10000); goat anti-mouse HRP (1:10000). After a series of washes, proteins were detected using the ECL Plus Western blotting chemiluminescent detection system (Pierce, UK), and signals were captured on Hyperfilm ECl autoradiography film (GE healthcare, UK). The exposure times for the detection of proteins were: K_ir_6.1, 10 sec; K_ir_6.2, 5 sec; SUR1, 10 min, SUR2, 15 min; GAPDH, 10 min for each blot after stripping and reprobing.

Primary and secondary antibodies were sourced from the following companies: anti K_ir_6.1 (Alomone Labs, Israel), anti K_ir_6.2, anti SUR1 and anti GAPDH (Millipore, UK), anti SUR2/ABCC9 (Abcam, UK), goat anti-rabbit HRP (Cell Signaling Technology, UK) and goat anti-mouse HRP (Millipore, UK).
